# Supplementary figures and images for: Formin-like 1β phosphorylation at S1086 is necessary for secretory polarized traffic of exosomes at the immune synapse in Jurkat T lymphocytes
Source: eLife. 2024 Oct 31;13:RP96942. doi: 10.7554/eLife.96942 (PMC11527432; doi:10.7554/eLife.96942)

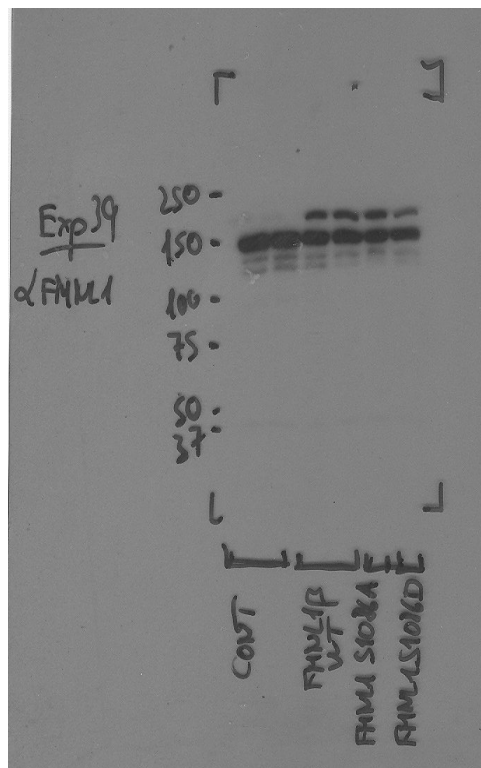

FMNL1 high-actin

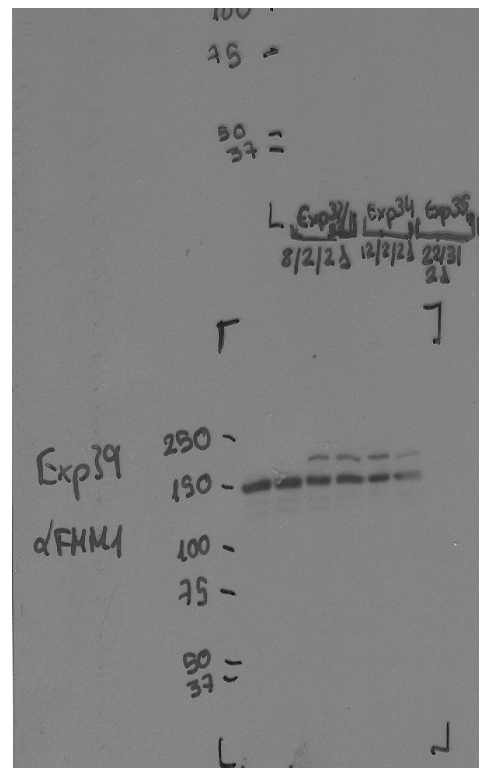

FMNL1 low-actin

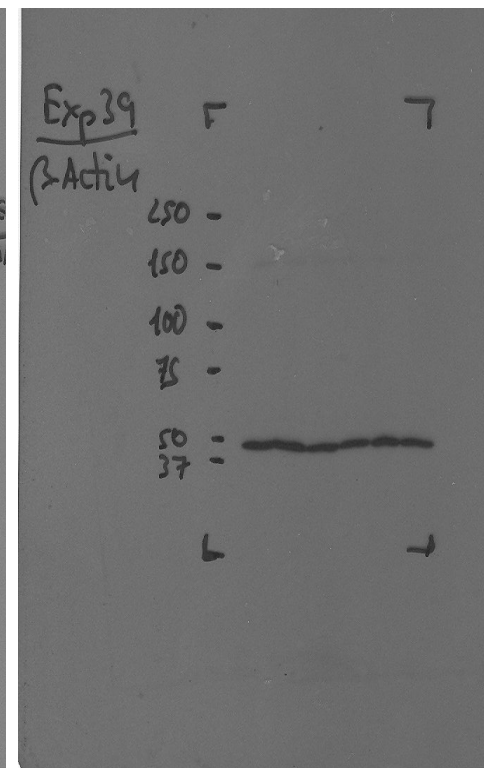

beta-actin

Supplement: Figure 2—source data 1. [file elife-96942-fig2-data1.zip › Figure 2_source data 1.pdf]

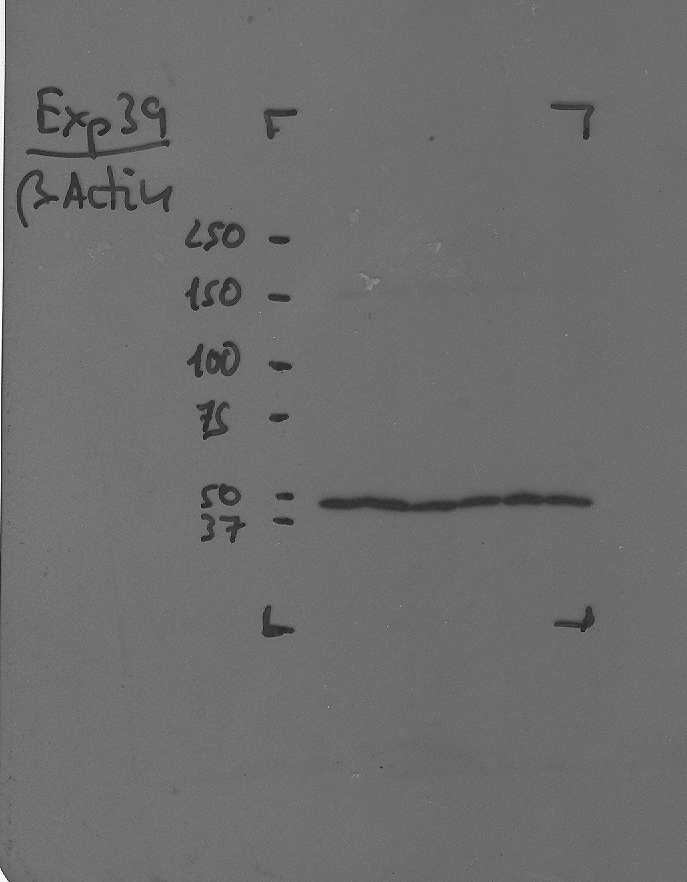

Supplement: Figure 2—source data 2. [file elife-96942-fig2-data2.zip › Figure 2_source data 2 tif/Bactin Exp 39_bajo 2024.tif]

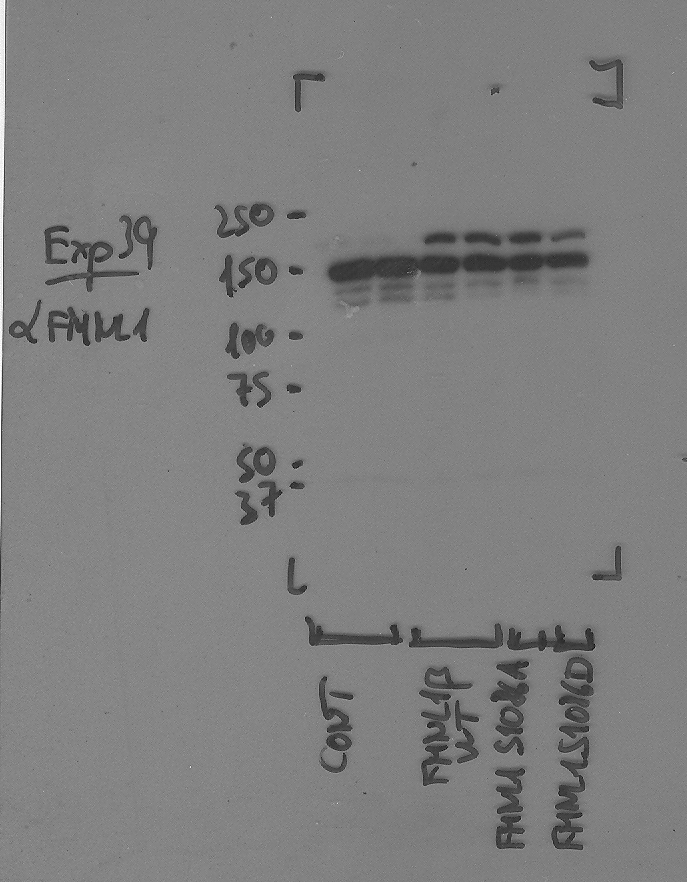

Supplement: Figure 2—source data 2. [file elife-96942-fig2-data2.zip › Figure 2_source data 2 tif/FMNL1 Exp 39_alto 2024.tif]

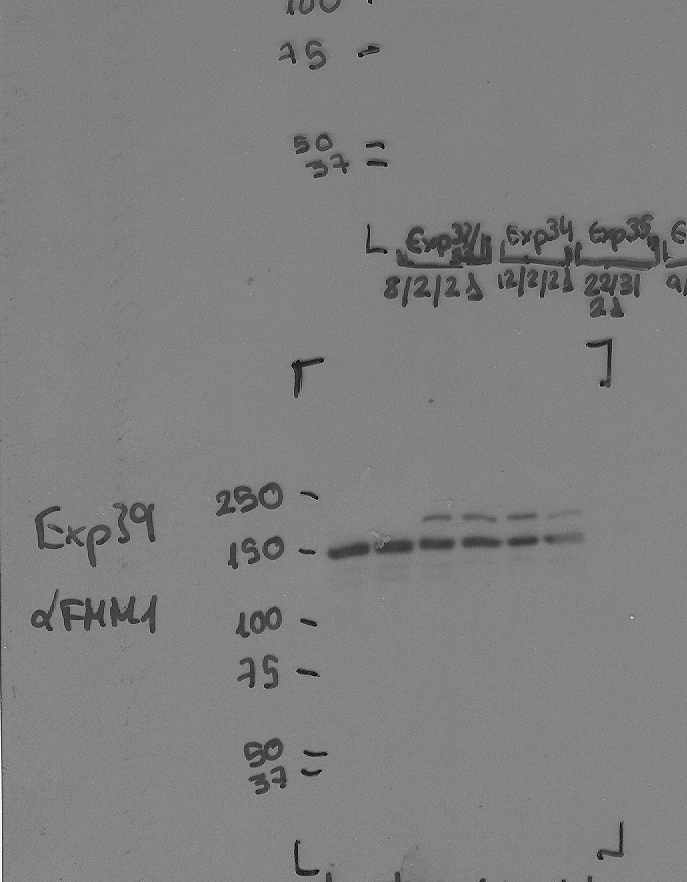

Supplement: Figure 2—source data 2. [file elife-96942-fig2-data2.zip › Figure 2_source data 2 tif/FMNL1 Exp 39_bajo 2024 001.tif]

# Anti-Phospho-Ser PKC substrate

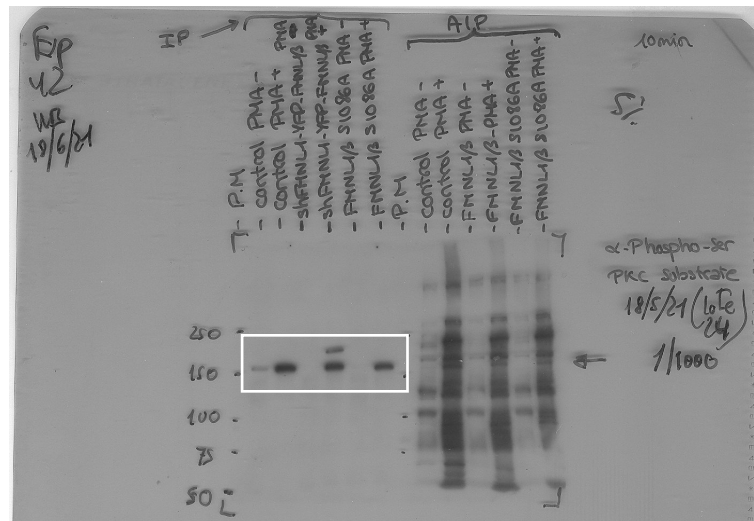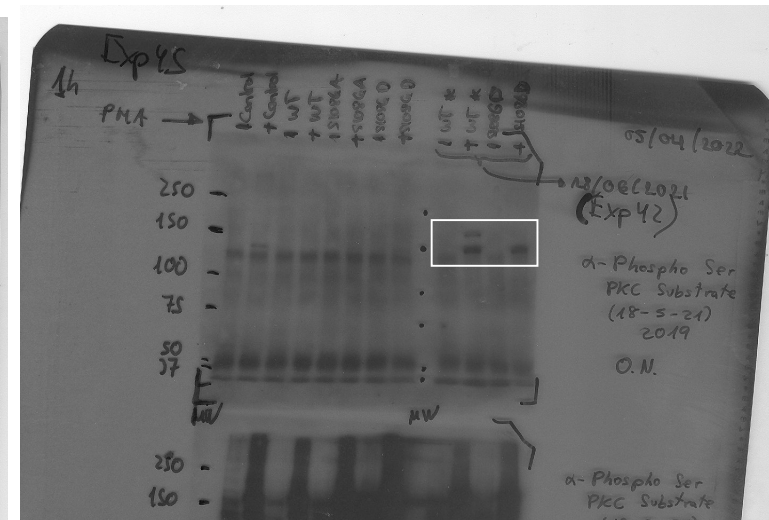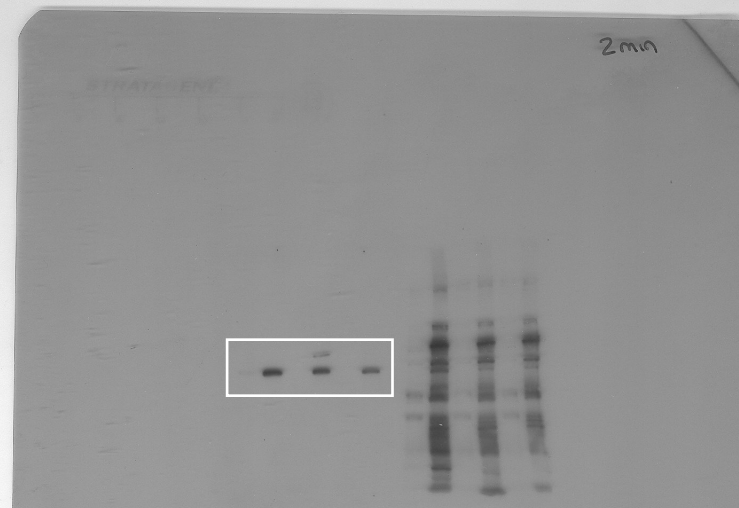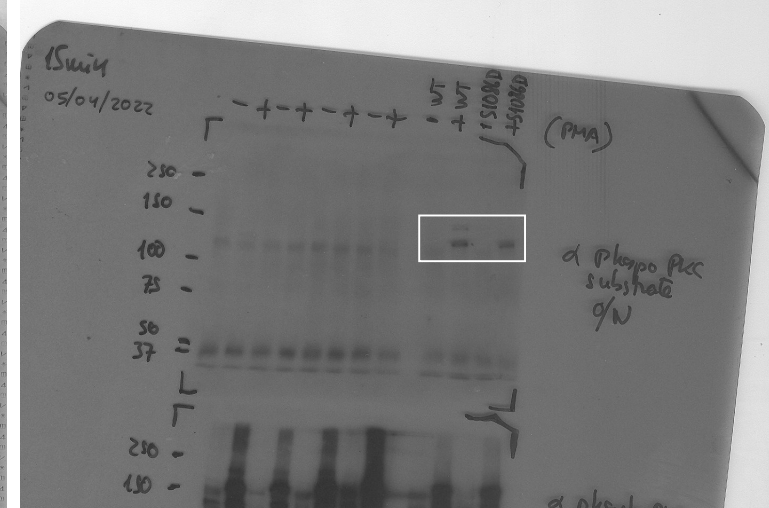

# Anti-FMNL1

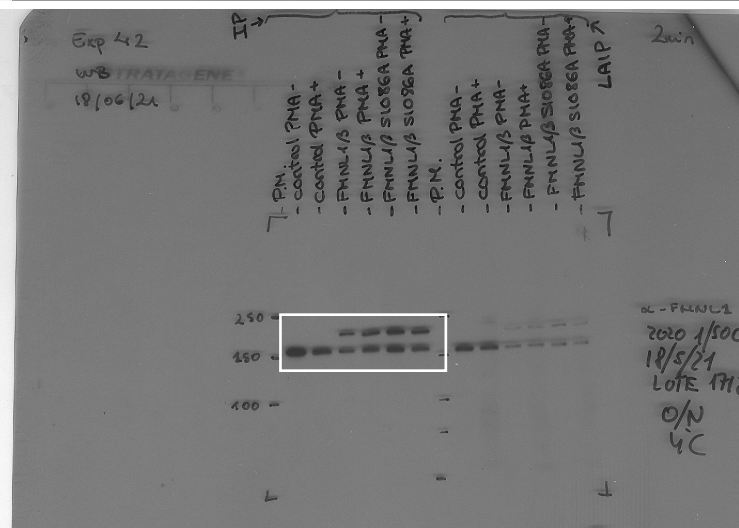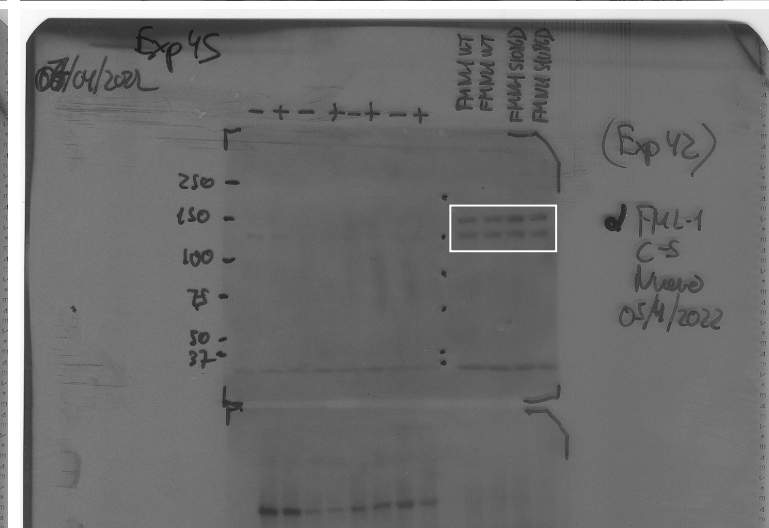

Supplement: Figure 3—source data 1. — White rectangles label the 6 cropped panels used in Figure 3. [file elife-96942-fig3-data1.zip › Figure 3_source data 1.pdf]

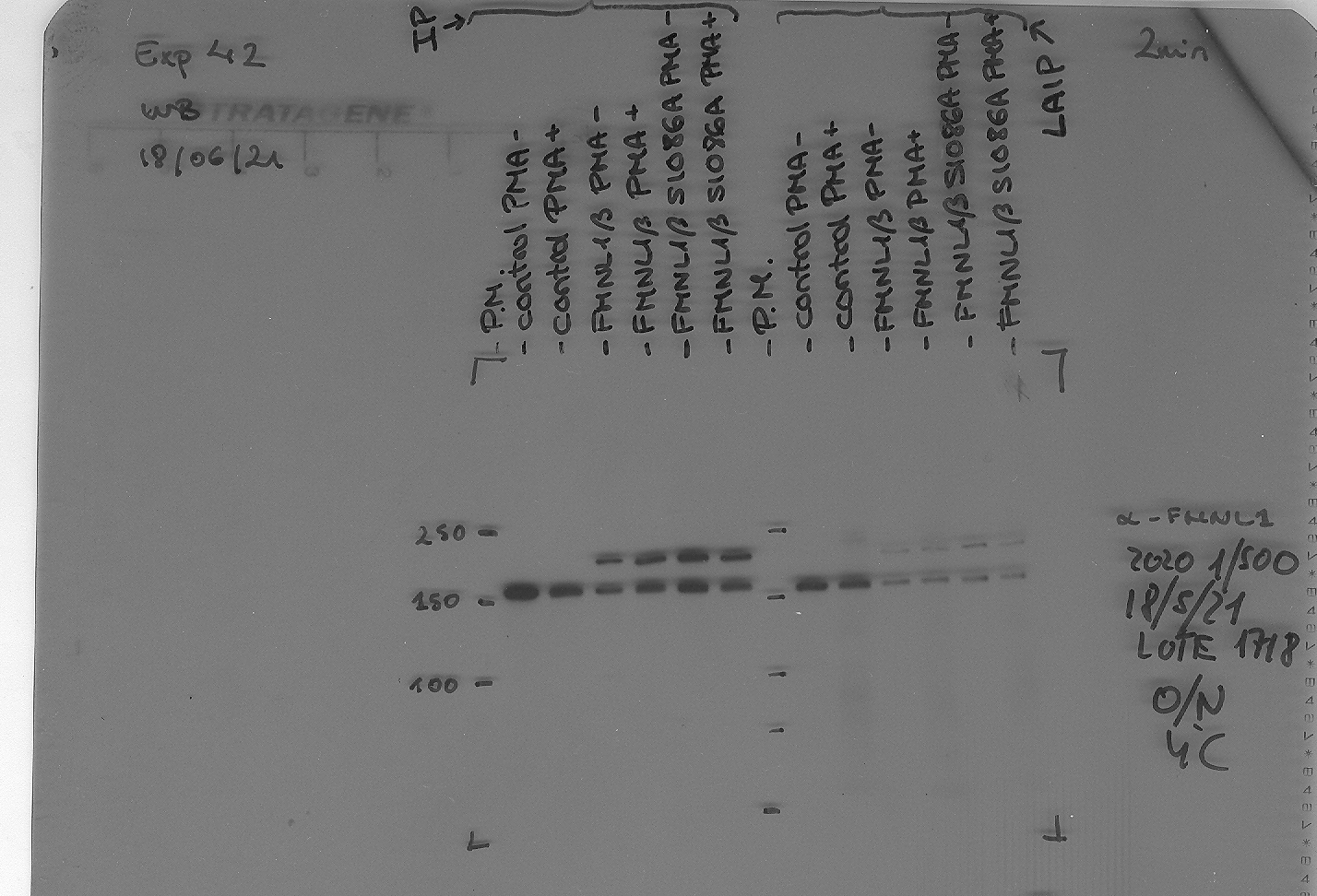

Supplement: Figure 3—source data 2. [file elife-96942-fig3-data2.zip › Figure 3_source data 2 tif/FMNL1 Exp 42_alto.tif]

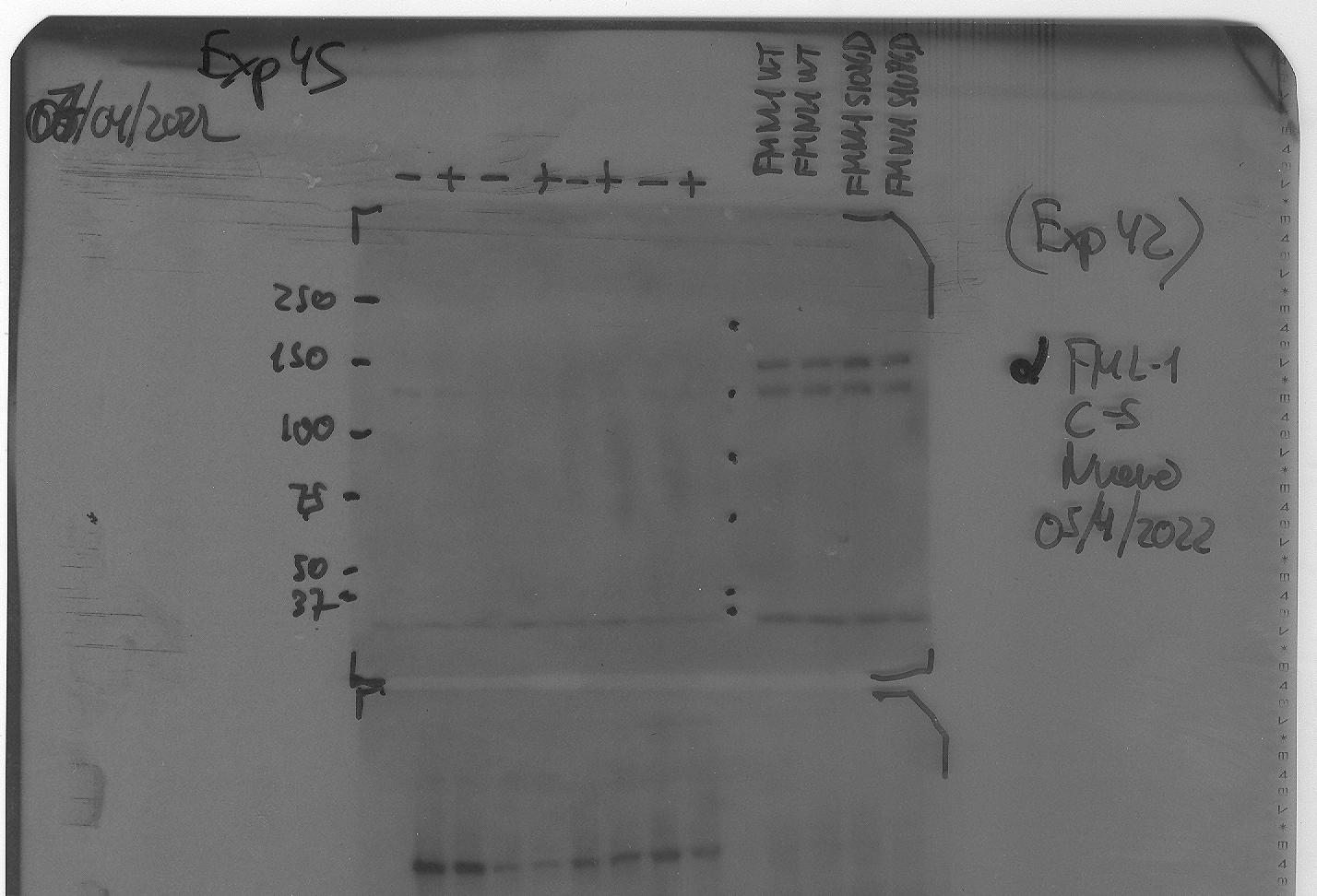

Supplement: Figure 3—source data 2. [file elife-96942-fig3-data2.zip › Figure 3_source data 2 tif/fmnl1exp 45bajo 001.tif]

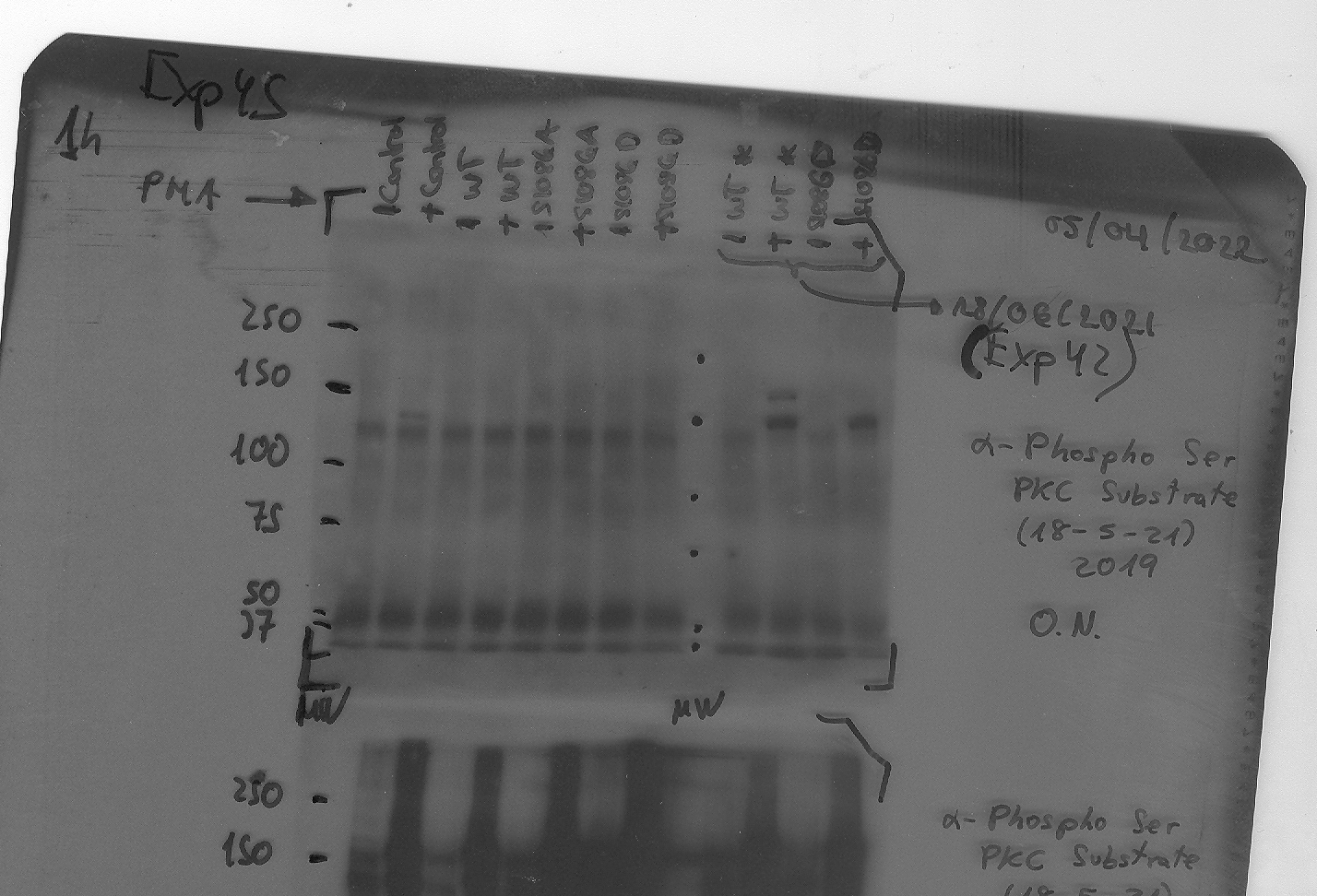

Supplement: Figure 3—source data 2. [file elife-96942-fig3-data2.zip › Figure 3_source data 2 tif/phospho exp 45alto.tif]

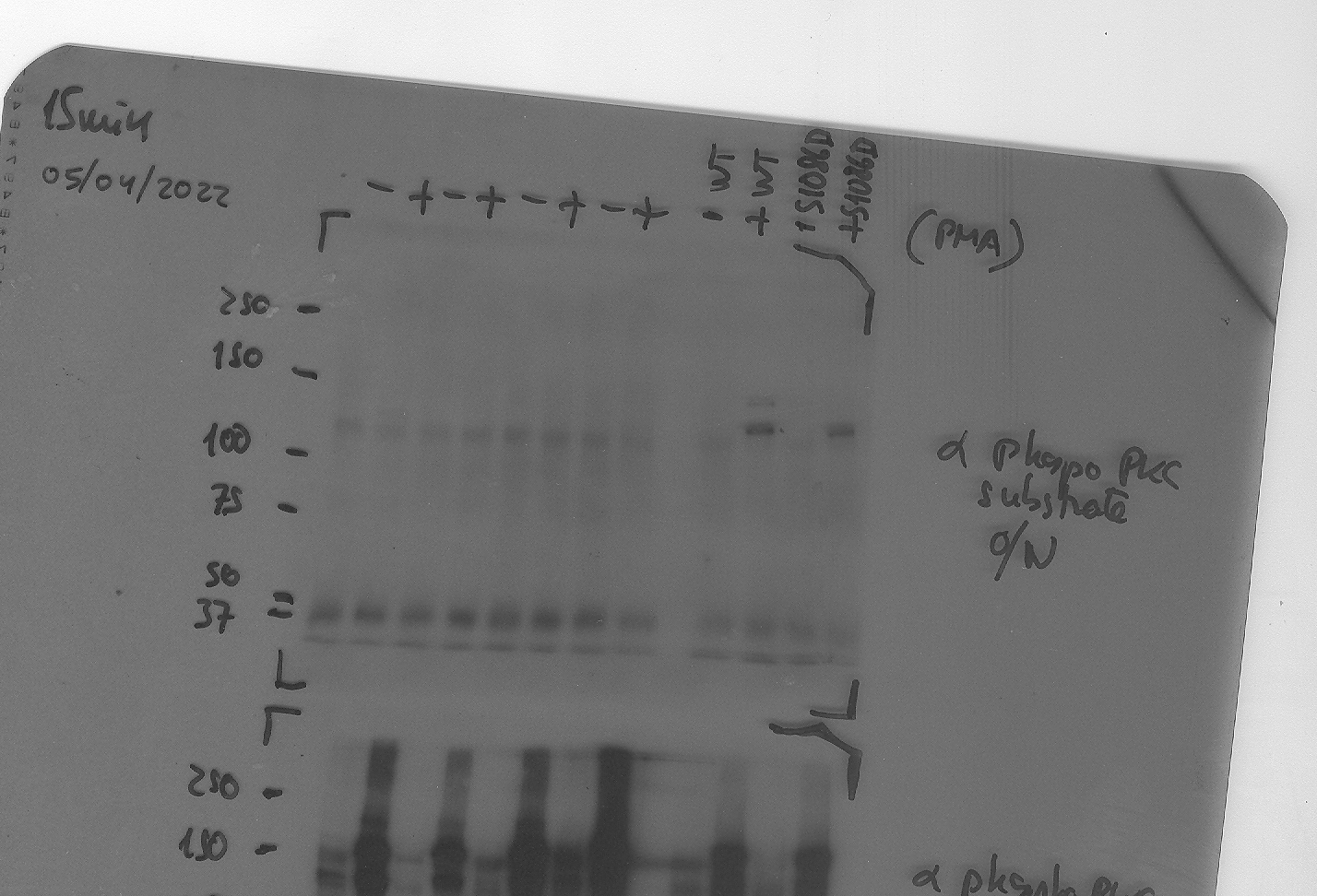

Supplement: Figure 3—source data 2. [file elife-96942-fig3-data2.zip › Figure 3_source data 2 tif/phospho exp 45bajo.tif]

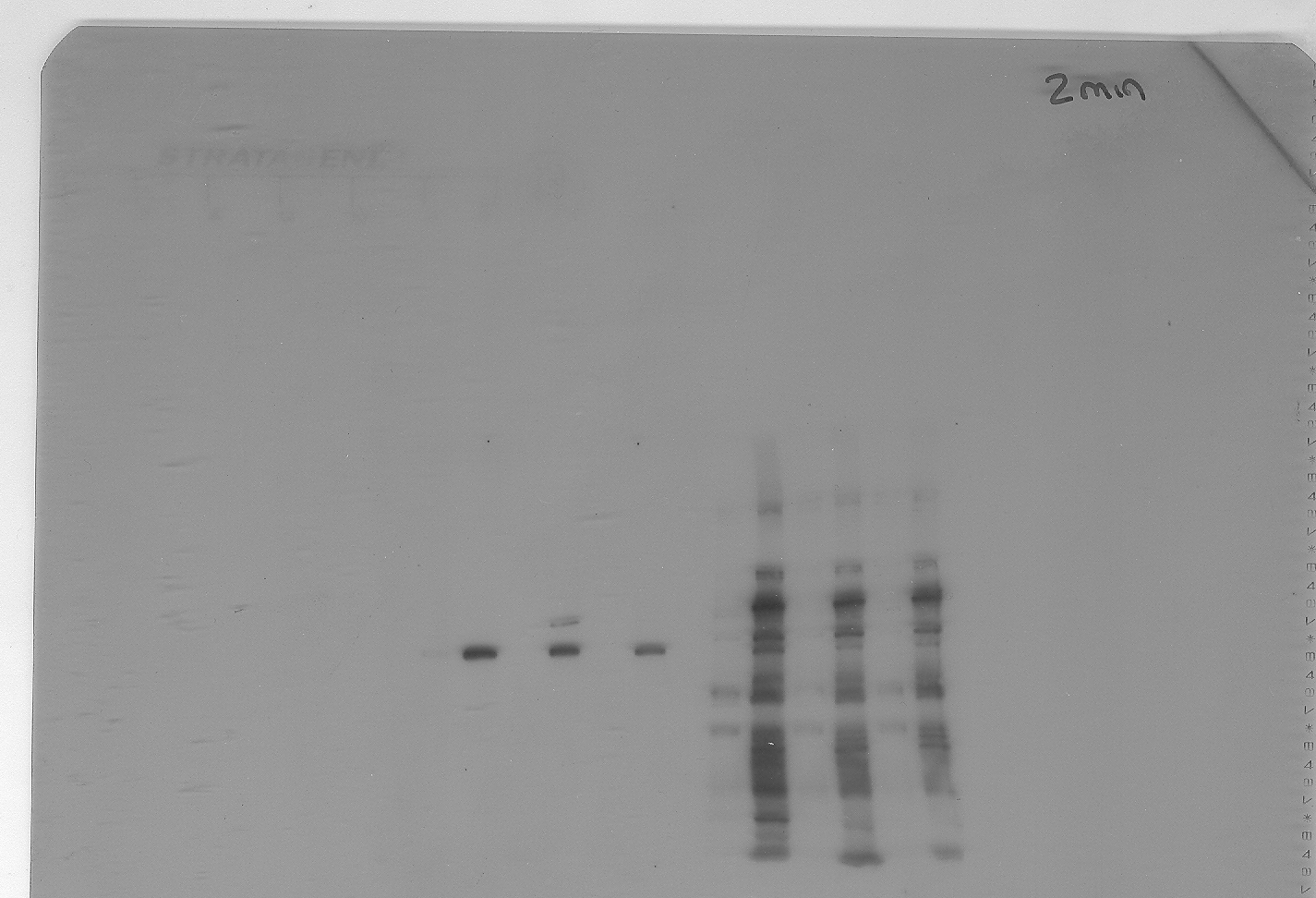

Supplement: Figure 3—source data 2. [file elife-96942-fig3-data2.zip › Figure 3_source data 2 tif/phospho PKC Exp 42_bajo.tif]

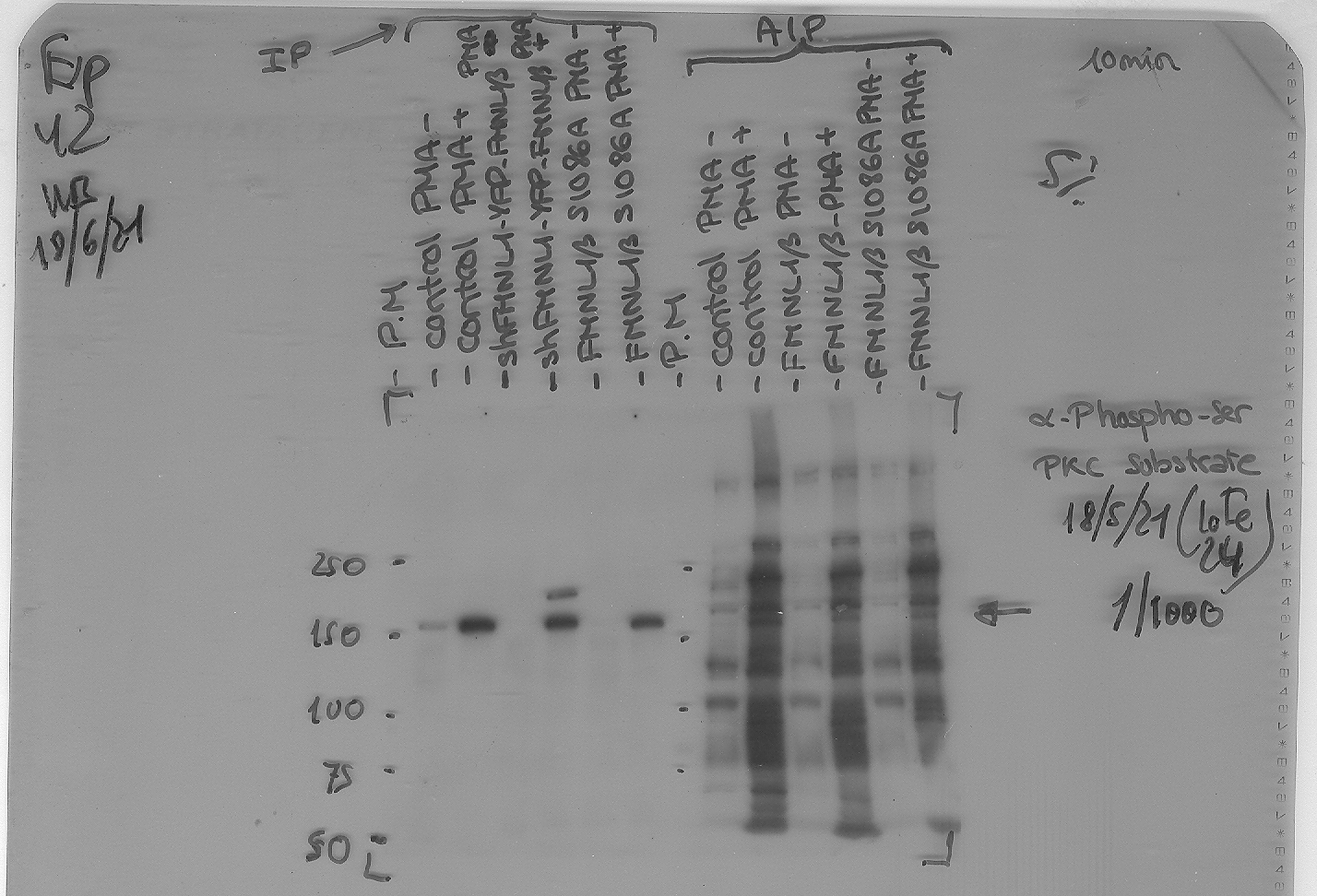

Supplement: Figure 3—source data 2. [file elife-96942-fig3-data2.zip › Figure 3_source data 2 tif/phospho PKC Expp 42_alto.tif]

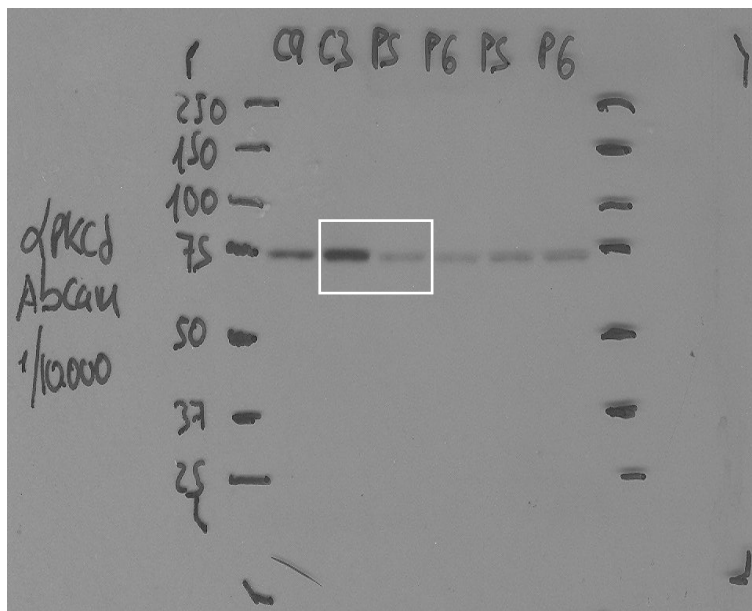

PKCdelta

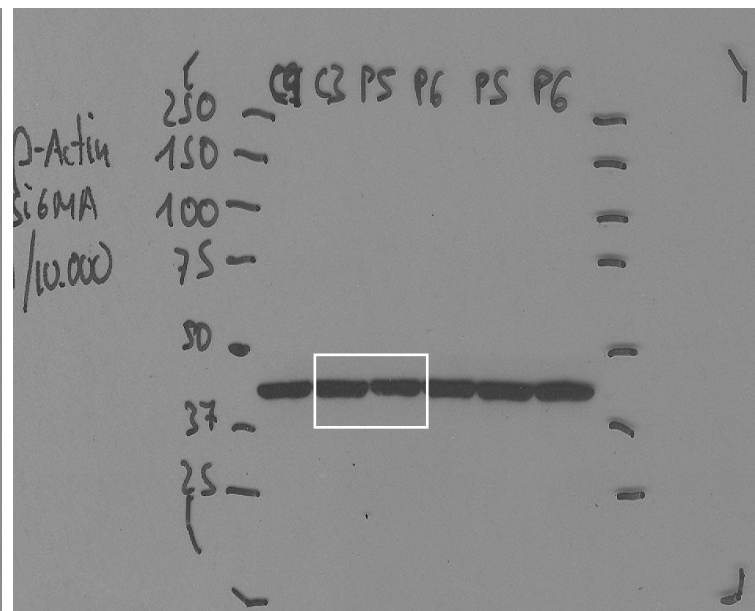

beta-actin

Supplement: Figure 6—source data 1. — White rectangles label the 2 cropped panels used in Figure 6A. [file elife-96942-fig6-data1.zip › Figure 6_source data 1.pdf]

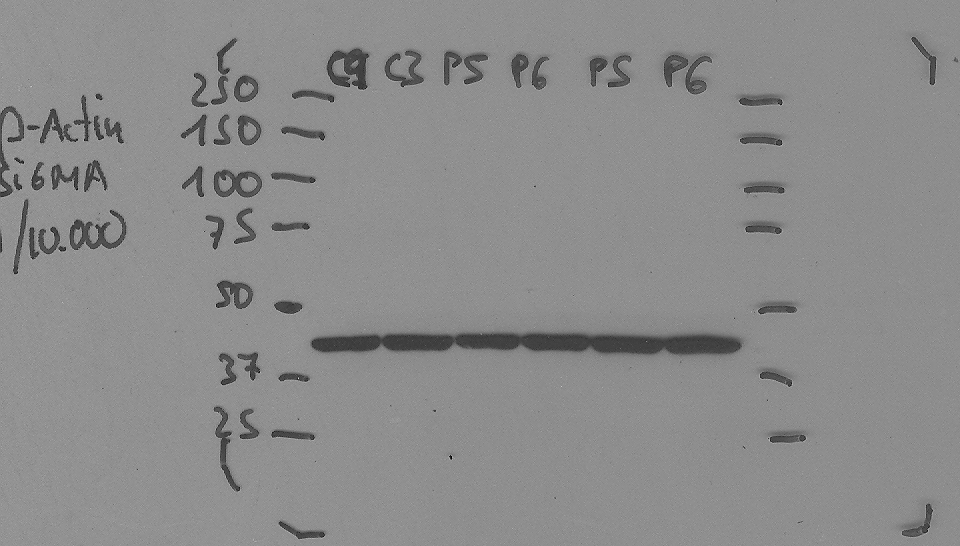

Supplement: Figure 6—source data 2. [file elife-96942-fig6-data2.zip › Figure 6_source data 2 tif/Bactina Alba 19_02_2013.tif]

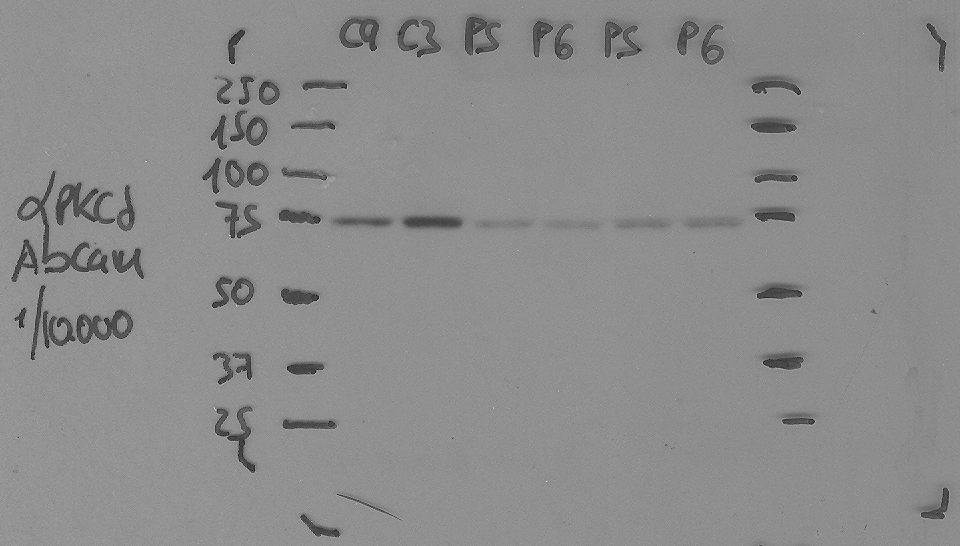

Supplement: Figure 6—source data 2. [file elife-96942-fig6-data2.zip › Figure 6_source data 2 tif/PKCdelta Alba 19_02_2013.tif]

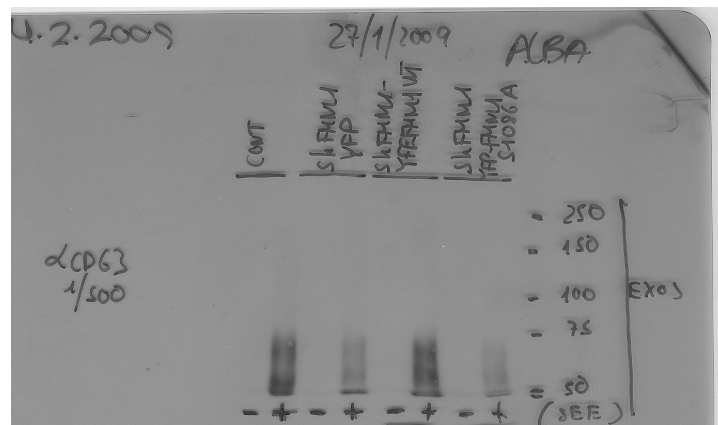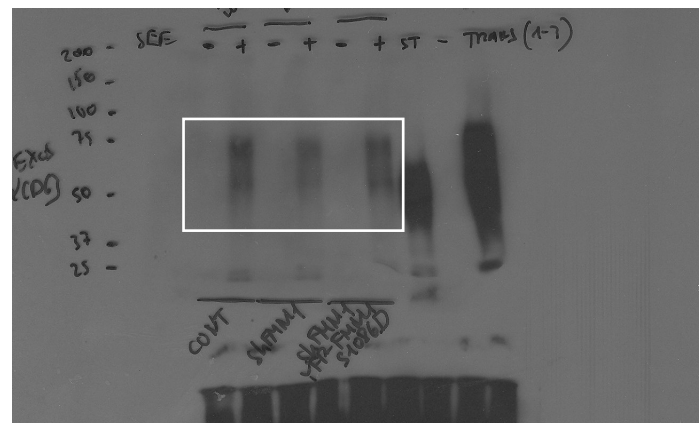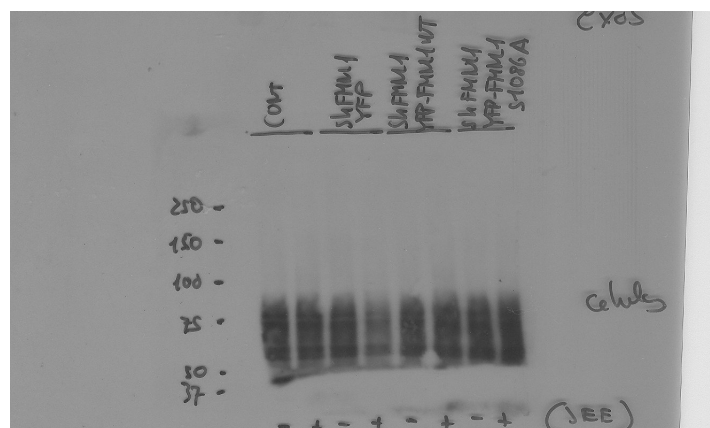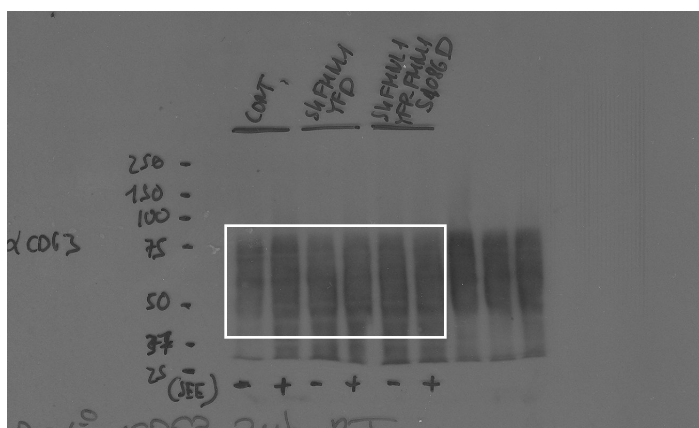

CD63

Supplement: Figure 9—source data 1. — White rectangles label the cropped panels used in Figure 9B. [file elife-96942-fig9-data1.zip › Figure 9_source data 1.pdf]

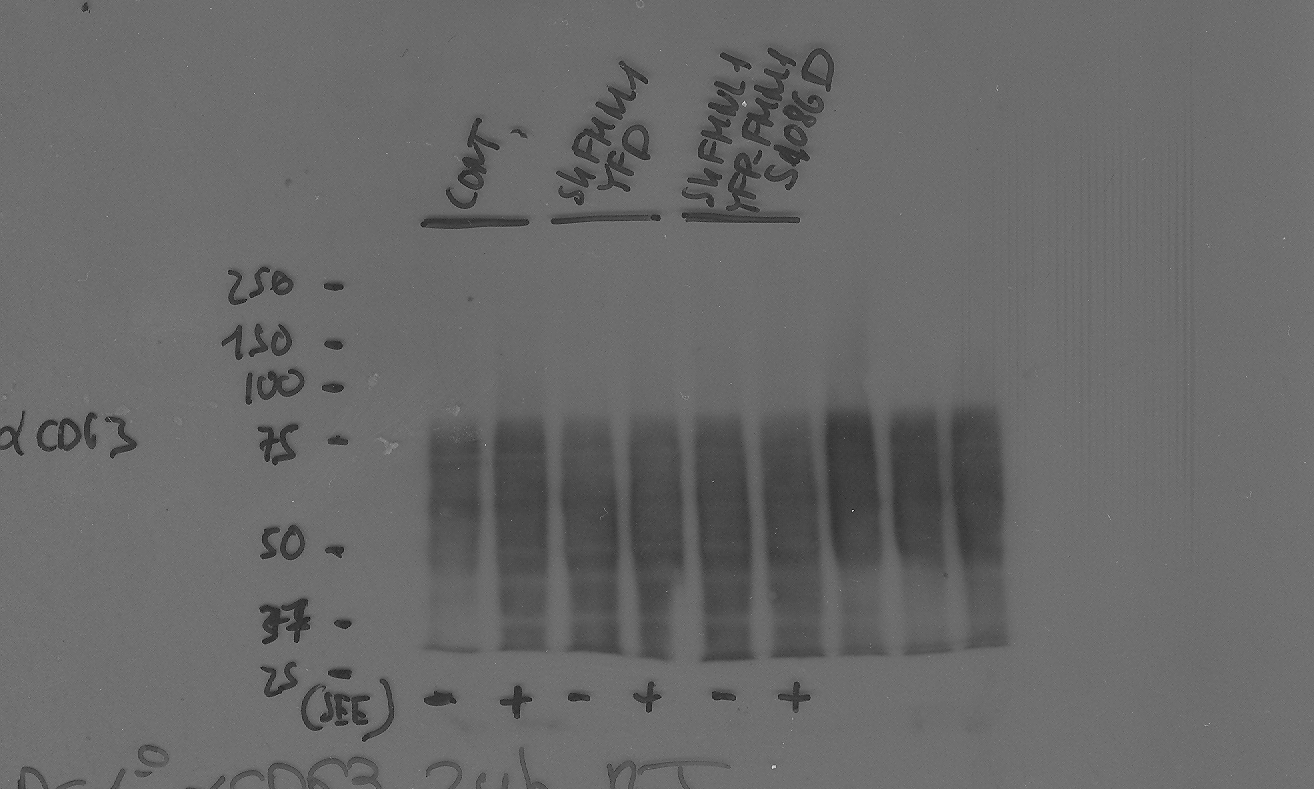

Supplement: Figure 9—source data 2. [file elife-96942-fig9-data2.zip › Figure 9_source data 2 tif/Cd63 cells Alba 15_12_2009.tif]

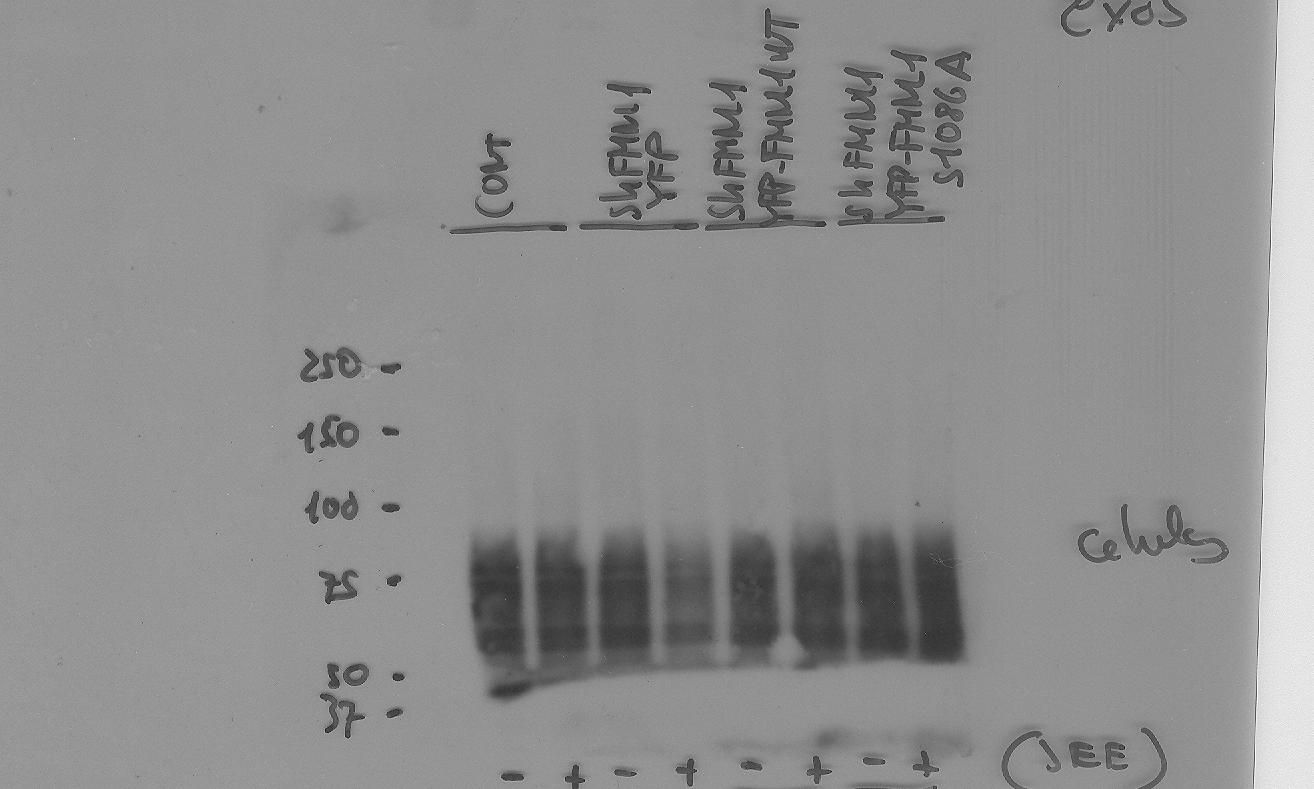

Supplement: Figure 9—source data 2. [file elife-96942-fig9-data2.zip › Figure 9_source data 2 tif/Cd63 cells Alba 27_01_2009.tif]

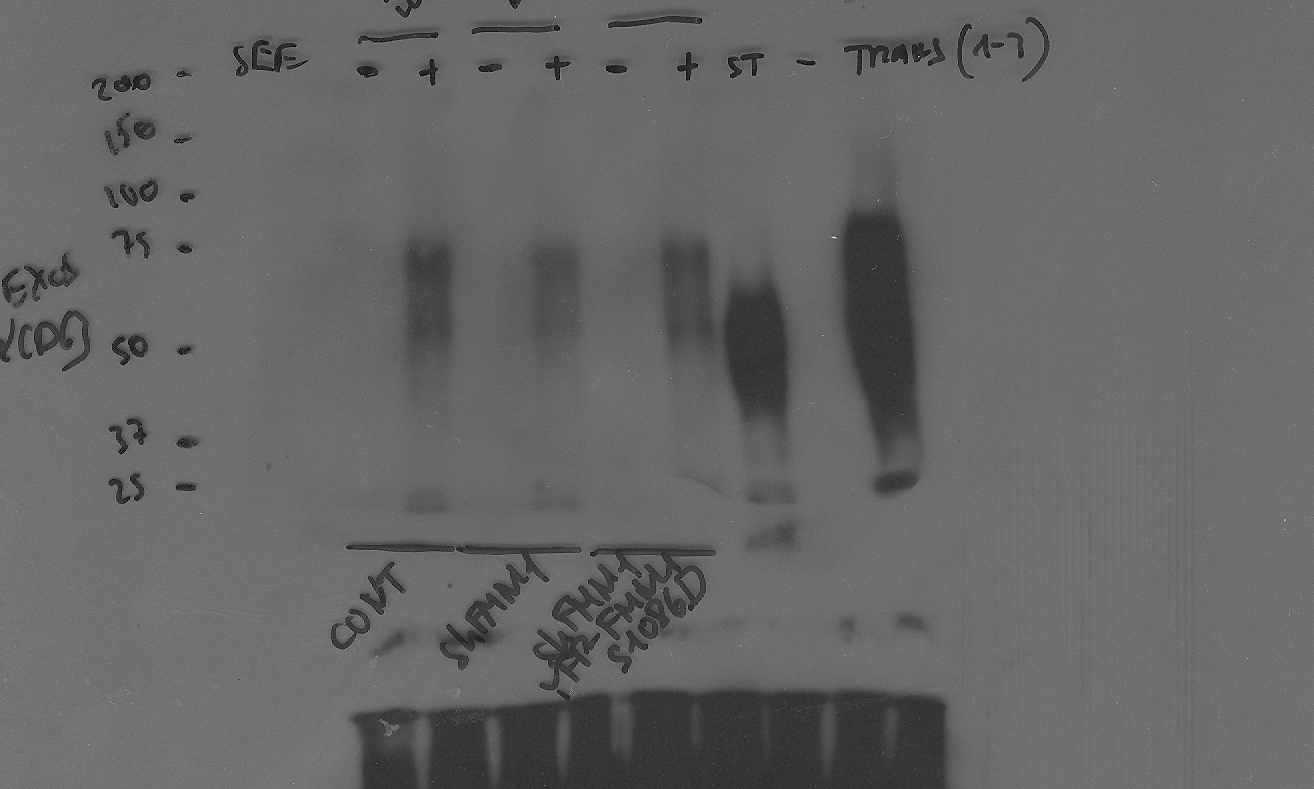

Supplement: Figure 9—source data 2. [file elife-96942-fig9-data2.zip › Figure 9_source data 2 tif/Cd63 exos Alba 15_12_2008.tif]

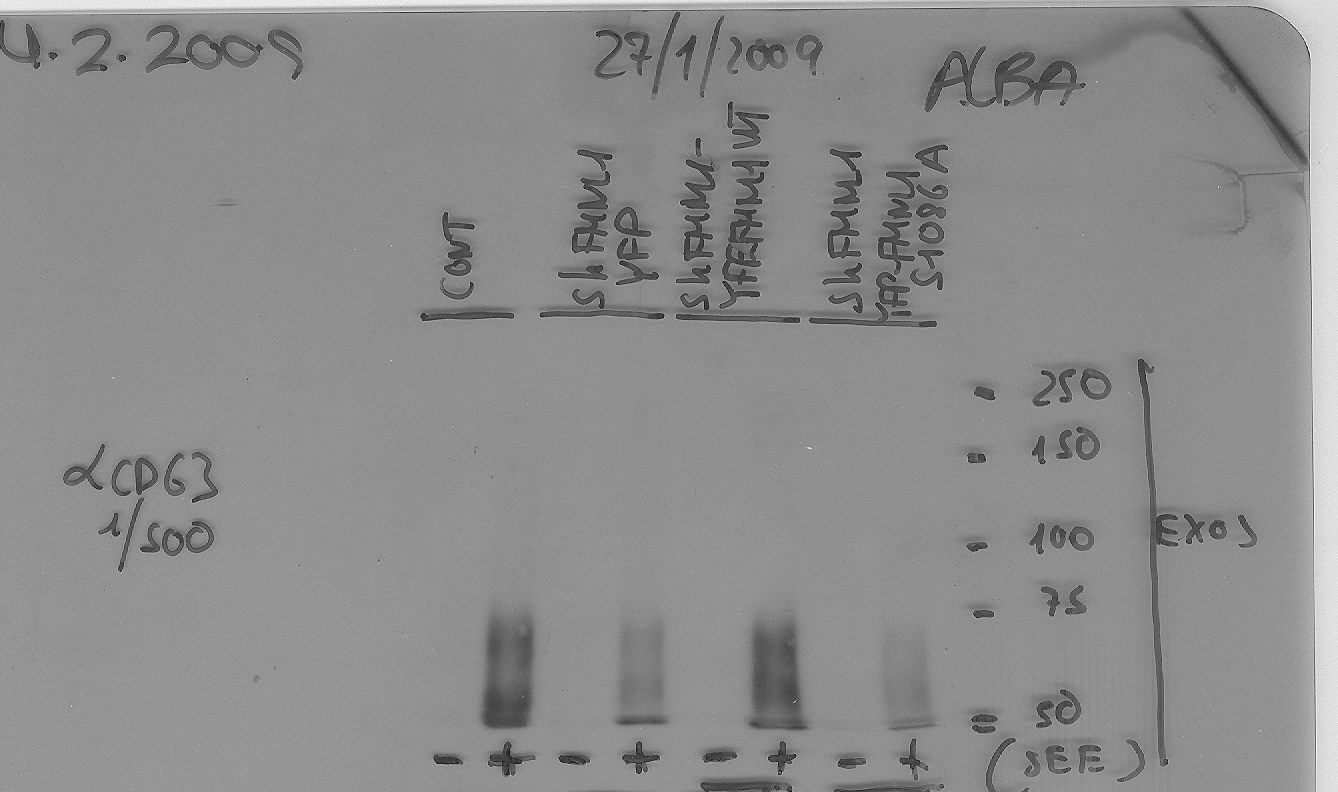

Supplement: Figure 9—source data 2. [file elife-96942-fig9-data2.zip › Figure 9_source data 2 tif/Cd63 exos Alba 27_01_2009CRP.tif]
