# Supplementary material for: Formin-like 1β phosphorylation at S1086 is necessary for secretory polarized traffic of exosomes at the immune synapse in Jurkat T lymphocytes
Source: eLife. 2024 Oct 31;13:RP96942. doi: 10.7554/eLife.96942 (PMC11527432; doi:10.7554/eLife.96942)
Supplement: Source data 1. [file elife-96942-data1.docx]

P-VALUES AFTER APPLYING TUKEY'S METHOD (POST HOC) TO THE ONE-WAY ANOVA

**Fig. 4**

**MTOC PI**

Control vs. shFMNL1 (0.0411)

Control vs. FMNL1βWT (0.7669)

Control vs. FMNL1βS1086A (0.0021)

Control vs. FMNL1βS1086D (0.9817)

shFMNL1 vs. FMNL1βWT (0.6026)

shFMNL1 vs. FMNL1βS1086A (0.9844)

shFMNL1 vs. FMNL1βS1086D (0.0223)

FMNL1βWT vs. FMNL1βS1086A (0.2328)

FMNL1βWT vs. FMNL1βS1086D (0.5428)

FMNL1βS1086A vs. FMNL1βS1086D (0.0014)

**MVB PI**

Control vs. shFMNL1 (0.0338)

Control vs. FMNL1βWT (0.6370)

Control vs. FMNL1βS1086A (0.0065)

Control vs. FMNL1βS1086D (0.9675)

shFMNL1 vs. FMNL1βWT (0.3750)

shFMNL1 vs. FMNL1βS1086A (>0.9999)

shFMNL1 vs. FMNL1βS1086D (0.1877)

FMNLβWT vs. FMNL1βS1086A (0.2036)

FMNL1βWT vs. FMNL1βS1086D (0.9757)

FMNL1βS1086A vs. FMNL1βS1086D (0.0834)

**Fig. 5**

(P5) Control vs. shFMNL1 (>0.9999)

(P5) Control vs. FMNL1βWT (0.9994)

(P5) Control vs. FMNL1βS1086A (>0.9999)

(P5) Control vs. FMNL1βS1086D (0.9983)

(P5) Control vs. Control (C3) (0.3473)

(P5) Control vs. shFMNL1 (C3) (0.5803)

(P5) shFMNL1 vs. FMNL1βWT (0.9934)

(P5) shFMNL1 vs. FMNL1βS1086A (0.9998)

(P5) shFMNL1 vs. FMNL1Sβ1086D (0.9885)

(P5) shFMNL1 vs. Control (C3) (0.3371)

(P5) shFMNL1 vs. shFMNL1 (C3) (0.9035)

(P5) FMNL1βWT vs. FMNL1βS1086A (>0.9999)

(P5) FMNL1βWT vs. FMNL1βS1086D (>0.9999)

(P5) FMNL1βWT vs. Control (C3) (0.8498)

(P5) FMNL1βWT vs. shFMNL1 (C3) (0.4507)

(P5) FMNL1βS1086A vs. FMNL1βS1086D (C3) (0.9997)

(P5) FMNL1βS1086A vs. Control (C3) (0.6279)

(P5) FMNL1βS1086A vs. shFMNL1 (C3) (0.6980)

(P5) FMNL1βS1086D vs. Control (C3) (0.9136)

(P5) FMNL1βS1086D vs. shFMNL1 (C3) (0.4276)

Control (C3) vs. shFMNL1 (C3) (0.0010)

**Fig. 7**

Control vs. shFMNL1 (0.0693)

Control vs. FMNL1βWT (0.9918)

Control vs. FMNL1βS1086A (0.0979)

Control vs. FMNL1βS1086D (0.9511)

shFMNL1 vs. FMNL1βWT (0.0346)

shFMNL1 vs. FMNL1βS1086A (>0.9999)

shFMNL1 vs. FMNL1βS1086D (0.0272)

FMNL1βWT vs. FMNL1βS1086A (0.051)

FMNL1βWT vs. FMNL1βS1086D (0.9982)

FMNL1βS1086A vs. FMNL1βS1086D (0.0389)

**Figure 2- figure supplement 1**

Control vs. shFMNL1 (0.0628)

Control vs. FMNL1βWT (0.0001)

Control vs. FMNL1βS1086A (<0.0001)

Control vs. FMNL1βS1086D (<0.0001)

shFMNL1 vs. FMNL1βWT (<0.0001)

shFMNL1 vs. FMNL1βS1086A (<0.0001)

shFMNL1 vs. FMNL1βS1086D (<0.0001)

FMNL1βWT vs. FMNL1βS1086A (0.7496)

FMNL1βWT vs. FMNL1βS1086D (0.0138)

FMNL1βS1086A vs. FMNL1βS1086D (0.2089)

**Figure 4- figure supplement 1**

Control (C3) vs. Control (C3) no SEE (0.0176)

Control (C3) vs. shFMNL1 (C3) (0.1057)

Control (C3) vs. Control (P5) (0.1813)

Control (C3) vs. Control (P5) no SEE (0.0021)

Control (C3) vs. shFMNL1 (P5) (0.2056)

Control (C3) sin SEE vs. shFMNL1 (C3) (>0.9999)

Control (C3) sin SEE vs. Control (P5) (0.9850)

Control (C3) sin SEE vs. Control (P5) no SEE (0.7975)

Control (C3) sin SEE vs. shFMNL1 (P5) (0.9998)

shFMNL1 (C3) vs. Control (P5) (0.9979)

shFMNL1 (C3) vs. Control (P5) no SEE (0.7907)

shFMNL1 (C3) vs. shFMNL1 (P5) (>0.9999)

Control (P5) vs. Control (P5) no SEE (0.4791)

Control (P5) vs. shFMNL1 (P5) (0.9997)

Control (P5) no SEE vs. shFMNL1 (P5) (0.7572)

**Figure 2- figure supplement 2**

**MTOC PI**

Control vs. shFMNL1 (0.0005)

Control vs. FMNL1βWT (0.1230)

Control vs. FMNL1βS1086A (0.0126)

Control vs. FMNL1βS1086D (0.6577)

shFMNL1 vs. FMNL1βWT (0.5336)

shFMNL1 vs. FMNL1βS1086A (0.9967)

shFMNL1 vs. FMNL1βS1086D (0.2372)

FMNL1βWT vs. FMNL1βS1086A (0.5909)

FMNL1βWT vs. FMNL1βS1086D (0.9575)

FMNL1βS1086A vs. FMNL1βS1086D (0.3206)

**MVB PI**

Control vs. shFMNL1 (0.0482)

Control vs. FMNL1βWT (>0.9999)

Control vs. FMNL1βS1086A (0.1643)

Control vs. FMNL1βS1086D (>0.9999)

shFMNL1 vs. FMNL1βWT (0.0381)

shFMNL1 vs. FMNL1βS1086A (0.8054)

shFMNL1 vs. FMNL1βS1086D (0.0672)

FMNL1βWT vs. FMNL1βS1086A (0.1264)

FMNL1βWT vs. FMNL1βS1086D (>0.9999)

FMNL1βS1086A vs. FMNL1βS1086D (0.2300)
